# Pleomorphic adenoma of the tongue: A case report

**DOI:** 10.1097/MD.0000000000033348

**Published:** 2023-03-24

**Authors:** Yuanzi Liang, Dong Bai, Wenting Liu, Zhiqun Wang

**Affiliations:** a Department of Radiology, Aerospace Center Hospital, Beijing, China; b Department of Pathology, Aerospace Center Hospital, Beijing, China.

**Keywords:** case report, oncology, pleomorphic adenoma, tongue

## Abstract

Salivary gland tumors account for approximately 3% of all tumors, most of which are benign, with pleomorphic adenomas being the most common, occurring mostly in middle-aged women, mostly originating from the major salivary glands and, to a lesser extent, from the minor salivary glands, with the tongue being a very rare site of occurrence. To date, case reports of pleomorphic adenoma at the root of the tongue are also rare.

**Patient concerns:**

A 56-year-old male patient with no obvious cause of foreign body sensation in the pharynx, sputum, no pain, no blood in the sputum, no dysphagia, and no difficulty in swallowing and breathing, which was significantly aggravated in the past 2 weeks, with difficulty in swallowing, breath-holding on lying down.

**Diagnoses:**

computed tomography and magnetic resonance imaging revealed a soft tissue mass at the root of the left tongue, which involved the tongue body in the forward direction. Electronic laryngopharyngoscopy showed a left-sided tongue root mass with a poorly smooth mucosa, covered with a mucous white pseudomembrane and a localized brownish-black crust without active bleeding. The final pathological findings showed a pleomorphic adenoma.

**Interventions:**

Postoperative symptomatic treatment was given, and the patient recovered well. Eight days after surgery, the patient was discharged from the hospital, and the pharyngeal pain basically subsided at the time of discharge, with no fever and no pharyngeal discomfort. Postoperative laryngoscopy showed smooth mucosa of the pharyngeal cavity, good pseudomembrane formation in the operated area, no active bleeding, no purulent secretions, and normal blood routine on recheck. The medical advice after discharge was firstly, full rest for 1 week, secondly, continue the oral anti-inflammatory treatment, 1 week after the operation need to review the outpatient clinic, finally, if there are any uncomfortable symptoms, seek medical attention in time.

**Outcomes:**

At present, the patient has been followed up for half a year and has recovered well from the operation without any discomfort.

**Lessons:**

It is very rare to find a pleomorphic adenoma of the tongue, and it occurs mostly in middle-aged women. In clinical diagnosis, it is sometimes difficult to distinguish it from malignant tumor of the tongue.

## 1. Introduction

Pleomorphic adenomas were first described by Minssen in 1874 in Ahlbom monograph.^[1]^ Pleomorphic adenomas are neoplastic lesions containing tumorigenic epithelial tissue and mucous-like or cartilage-like tissue, with a pleomorphic and mixed histology, hence the name pleomorphic adenoma. Most of them originate from the major salivary glands and a few from the minor salivary glands,^[2]^ but lingual pleomorphic adenomas are very rare and the number of reported cases is limited. We report a rare case of pleomorphic adenoma of the tongue presenting as a tongue mass in a 56-year-old male patient and discuss its clinical features, imaging diagnosis and differential diagnosis and treatment.

## 2. Case presentation

**Figure 1.**
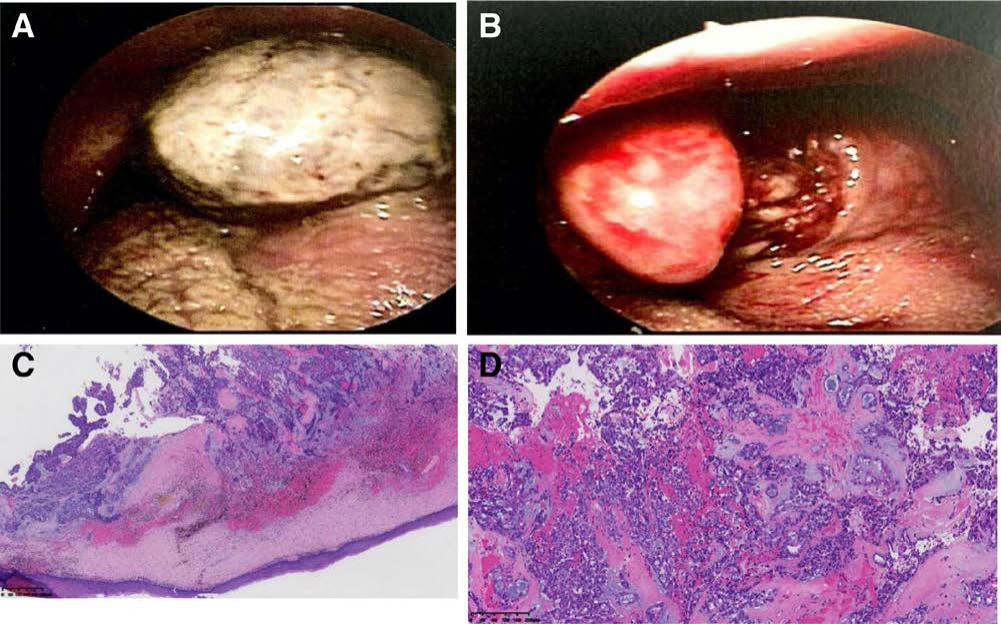
Left tongue root source elevated swelling, surface mucosa less than smooth, smudge-like mucous white pseudomembrane attachment, (A) brittle, easy to bleed, (B) at magnification 40x, the tumor was located in the submucosa, no obvious envelope was seen, and old hemorrhage was seen around the tumor, (C) at magnification 100x, the tumor was composed of short columnar and cuboidal epithelial and myoepithelial cells, and cartilaginous mucus-like stroma was seen in the interstitium, and (D).

**Figure 2.**
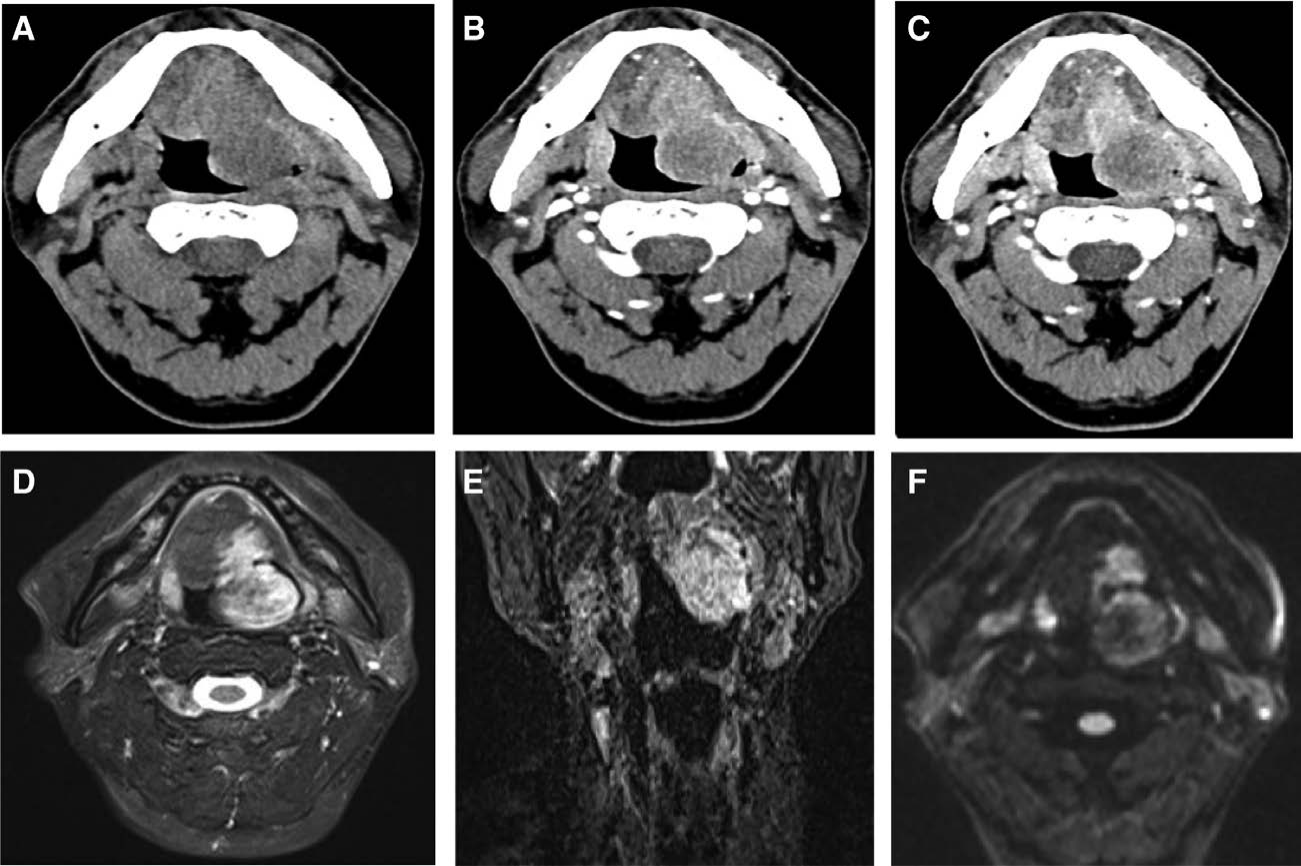
Pleomorphic adenoma of the left tongue CT scan, arterial phase and venous phase shows: irregular soft tissue density occupancy of the left tongue with heterogeneous density and progressive enhancement after enhancement, (A–C) axial compression lipid *T*2-weighted image sequence, (D) coronal compression lipid *T*2-weighted image sequence sequence, (E) Axial DWI sequence b = 1000mm/s^2^, and (F) shows: *T*2-weighted image sequence of the left tongue shows a heterogeneous obvious high signal occupancy, with linear low signal of each sequence visible at the edge and high signal on DWI. CT = computed tomography.

A 56-year-old male patient presented with a foreign body sensation in the throat with no apparent cause 3 months ago, sputum, no pain, no blood in the sputum, no dysphagia, no dyspnea, oral medication in the local clinic (specific unknown), the effect is not good, in the last 2 weeks the foreign body sensation in the throat significantly aggravated, with difficulty in swallowing, lying down to hold the breath, no pain and fever. past 35 years of smoking history and hypertension history for more than 1-month, irregular medication, blood pressure control is poor, had oral aspirin, now stopped, no special family genetic history. The result of physical examination showed chronic congestion of the mucosa of the pharyngeal cavity, narrowing of the pharyngeal cavity, and a spherical bulge at the left tongue root with an egg-sized swelling that blocked the pharyngeal cavity and moved with the tongue body. Electronic laryngopharyngoscopy (Fig. 1A and B) showed a swelling at the root of the left tongue with poorly smooth mucosa, covered with a mucous white pseudomembrane, and a localized brownish-black crust without active bleeding. Enhanced computed tomography (CT) of the neck showed an irregular soft tissue mass at the root of the tongue on the left side, measuring approximately 4.9 cm × 2.7 cm × 3.6 cm, with a CT value of 57 Hu and less uniform density, extending forward to the body of the tongue, and the enhancement scan was mildly heterogeneous, with progressive enhancement, no obvious enlarged lymph nodes in the neck, and no clear signs of destruction of adjacent bone. (Fig. 2A–C). Magnetic resonance imaging (MRI) showed irregular soft tissue signal was seen at the left tongue root, with slightly low signal in *T*1-weighted image, mixed high signal in *T*2-weighted image, linear low signal at the edges, extending towards the tongue body, and inhomogeneous high signal in diffusion weighted imaging (Fig. 2E and F), with local apparent diffusion coefficient signal reduction. Comprehensive imaging diagnosis: the left tongue root occupancy, involving the tongue body, considered benign possibility. A transendoscopic mass excision was performed, and an intraoperative mass was seen at the root of the left tongue with a brittle surface that bleeds easily when touched, and a tough mass border that forms an envelope. Postoperative pathology (Fig. 1C and D): grayish brown fragmented tissue, size about 4.5 cm × 4 cm × 1.5 cm. The tumor was located in the submucosa, and old hemorrhage was seen around the tumor. The tumor was composed of dwarf columnar and cuboidal epithelial and myoepithelial cells, and a cartilaginous mucus-like stroma was seen in the interstitium. Immunohistochemistry: CK7 (+), CK5/6 (partial +), S-100 (+), Ki67 (+<5%), P53 (−), CK18 (+). Pathological diagnosis: pleomorphic adenoma of the tongue. The patient was returned to the intensive care unit after postoperative tracheal intubation. After 2 days, the patient was fully awakened and checked that there was no active bleeding in the oropharynx, the tracheal tube was removed, and the patient returned to the ward and was given intravenous anti-inflammatory, rehydration and other symptomatic treatment. The patient was discharged from the hospital 8 days after the operation. At the time of discharge, the pharyngeal pain basically subsided, no fever, no pharyngeal discomfort, stable vital signs, and postoperative laryngoscopy showed smooth mucosa of the pharyngeal cavity, medium pendulous, left The postoperative laryngoscopy showed that the mucosa of the pharyngeal cavity was smooth, the pendulous lobe was centered, the left side of the tongue root was mildly swollen, the pseudomembrane in the operated area was well formed, there was no active bleeding, no purulent discharge, and the routine blood leukocytes returned to normal. There are roughly 4 medical orders for this patient’s discharge, firstly, full rest for 1 week, secondly, continue the oral anti-inflammatory treatment, 1 week after the operation need to review the outpatient clinic, then continue the mouthwash, and finally, if there are any uncomfortable symptoms, please seek medical attention in time. At present, it has been followed up for 6 months, and the postoperative recovery is good without any discomfort.

## 3. Disscussion

Salivary gland tumors account for about 3% of all tumors, and most of them are benign, among which pleomorphic adenomas are the most common,^[3]^ pleomorphic adenomas are neoplastic lesions containing tumorigenic epithelial tissue and mucus-like or cartilage-like tissue, generally with an intact envelope, when no envelope or incomplete envelope is seen, it means that the tumor is aggressive and has a malignant tendency, with pleomorphic and mixed histology, so it is also called mixed Most tumors occur in middle-aged women, most of them originate from the major salivary glands, and a few from the minor salivary glands, and the minor salivary glands mostly involve the palate, followed by the lips,^[4]^ while the tongue is a very rare site of occurrence. Chaudhry et al^[5]^ found in a study of 1320 tumors that only 1% of salivary gland tumors originated in the tongue, and when lesions occurred in the tongue, the root of the tongue was the most common site, with a progressive decrease in incidence from posterior to anterior. To date, case reports of pleomorphic adenoma of the tongue root are also very rare, and according to previous case reports, dysphagia is the first symptom, which was confirmed by the study of Yoshihara T,^[6]^ and the present case presented with progressive foreign body sensation in the pharynx with dysphagia, and the clinical symptoms were consistent with the literature.

The most commonly used imaging methods for tongue pleomorphic adenoma are CT and MRI. CT can detect the adjacent bony changes and surrounding enlarged lymph nodes in time, while MRI can better define the scope of the tumor and the internal components of the lesion. Most pleomorphic adenomas have abundant mucus stroma and high signal in *T*2-weighted image sequence, but the *T*2-weighted image signal is not uniform due to the uneven distribution of epithelial tissue and mucus-like tissue. The tumor margins are clear and low signal foci are seen in each sequence, which is also consistent with old hemorrhage around the pathology and is a more characteristic manifestation of pleomorphic adenoma. CT enhancement scans are mildly heterogeneous with progressive enhancement, and this enhancement is due to the fact that pleomorphic adenoma is composed of glandular epithelial cells and myoepithelial cells, sometimes in the form of adenoid structures, which are sparsely vascularized and rich in mucus-like and cartilage-like mesenchyme, and the arteries on enhancement scans the contrast agent that enters the tumor at the early stage is less, but due to the slow elimination of contrast agent, the contrast agent stays in the tumor for a longer time. Differential diagnosis of tongue occupancy imaging: squamous cell carcinoma of the tongue: often occurs in middle-aged and elderly people, preferably at the edge of the tongue, with unclear borders, strong aggressiveness, and deep infiltration, obvious inhomogeneous enhancement, most early lymph node metastasis can be detected; lymphoma: MRI signal is uniform, with mild to moderate uniform enhancement, multiple enlarged lymph nodes can be seen in other areas; adenoid cystic carcinoma: more pieces in middle-aged and elderly people, preferably at the root of the tongue, imaging Soft tissue mass, equal or slightly hypointense, multiple cystic hypointense foci forming sieve-like changes, without pseudo-envelope, and uneven enhancement. The final diagnosis depends on the pathological findings.

Currently, the most common treatment for pleomorphic adenoma of the tongue is surgical excision, but the manner and extent of excision depends on individual circumstances, such as age, lesion size, location, and general condition of the patient. Surgical approaches include transoral, transcervical, midline transglottic, transpharyngeal, and lateral pharyngeal dissection, with care needed to protect the hypoglossal and lingual nerves, and complete transoral endoscopic excision of the mass in the patient in this article. Recurrence of this disease is uncommon and may be due to partial resection or multifocal origin of the tumor^[7]^

## 4. Conclusion

We report a rare case of pleomorphic adenoma of the tongue. Although pleomorphic adenomas can occur in minor salivary glands, tongue pleomorphic adenoma is quite rare and the number of reported cases is limited. When imaging reveals an encapsulated soft tissue density occupancy in the tongue, especially at the tongue root, with limited nuclear magnetic diffusion, and uneven progressive enhancement without surrounding enlarged lymph nodes, the possibility of a pleomorphic adenoma of the tongue needs to be considered. Adequate excision of the diseased tissue under direct microscopic surgery is curable in most cases.

## Author contributions

**Investigation:** Yuanzi Liang, Dong Bai, Wenting Liu.

**Supervision:** Yuanzi Liang, Dong Bai.

**Writing – original draft:** Yuanzi Liang, Dong Bai.

**Writing – review & editing:** Zhiqun Wang.
